# Supplementary material for: An open-source framework for large-scale, flexible evaluation of biomedical text mining systems
Source: J Biomed Discov Collab. 2008 Jan 29;3:1. doi: 10.1186/1747-5333-3-1 (PMC2276192; doi:10.1186/1747-5333-3-1)
Supplement: Additional file 1 — Evaluation platform source code. The additional file contains the software evaluation framework discussed in this paper. The most current release can be downloaded from SourceForge.net [1]. See the accompanying README file for instructions on installation and use. Also included in the distribution is a collection of UIMA wrappers for some commonly used BioNLP tools and annotated corpora. [file 1747-5333-3-1-S1.zip › bionlp_uima_repository-1.0.1/docs/api/src-html/edu/uchsc/ccp/uima/ae/nlp/ei/gene/LingPipeEntityTagger_AE.html]

```
001    /*
002     * Created on Sep 8, 2005
003     *
004     * TODO To change the template for this generated file go to
005     * Window - Preferences - Java - Code Style - Code Templates
006     */
007    package edu.uchsc.ccp.uima.ae.nlp.ei.gene;
008    
009    import java.io.IOException;
010    import java.util.HashSet;
011    import java.util.Iterator;
012    import java.util.List;
013    import java.util.Set;
014    
015    import org.apache.uima.analysis_engine.ResultSpecification;
016    import org.apache.uima.analysis_engine.annotator.AnnotatorConfigurationException;
017    import org.apache.uima.analysis_engine.annotator.AnnotatorContext;
018    import org.apache.uima.analysis_engine.annotator.AnnotatorContextException;
019    import org.apache.uima.analysis_engine.annotator.AnnotatorInitializationException;
020    import org.apache.uima.analysis_engine.annotator.AnnotatorProcessException;
021    import org.apache.uima.analysis_engine.annotator.JTextAnnotator_ImplBase;
022    import org.apache.uima.jcas.JCas;
023    
024    import edu.uchsc.ccp.uima.annotation.CCPDocumentInformation;
025    import edu.uchsc.ccp.uima.util.UIMA_Util;
026    import edu.uchsc.ccp.util.external.lingpipe.LingPipe_Util;
027    import edu.uchsc.ccp.util.nlp.annotation.AnnotationSet;
028    import edu.uchsc.ccp.util.nlp.annotation.Annotator;
029    import edu.uchsc.ccp.util.nlp.annotation.TextAnnotation;
030    
031    /**
032     * @author williamb Takes longer time to run the first time.. not sure why
033     */
034    public class LingPipeEntityTagger_AE extends JTextAnnotator_ImplBase {
035        private final boolean DEBUG = false;
036    
037        public static final String PARAM_LINGPIPE_ENTITY_MODEL_FILE = "entityModelFile";
038    
039        public static final String PARAM_LINGPIPE_ENTITY_TYPE = "entityType";
040        
041        private final Annotator annotator = new Annotator (new Integer(22), "","LingPipe","Alias-i");
042        private final AnnotationSet annotationSet = new AnnotationSet(-1, "Default Annotation Set","Default Annotation Set");
043    
044        public void initialize(AnnotatorContext ac) throws AnnotatorInitializationException, AnnotatorConfigurationException {
045            String lingPipeEntityModelFile;
046            String entityType;
047    
048            try {
049                lingPipeEntityModelFile = (String) ac.getConfigParameterValue(PARAM_LINGPIPE_ENTITY_MODEL_FILE);
050                entityType = (String) ac.getConfigParameterValue(PARAM_LINGPIPE_ENTITY_TYPE);
051            } catch (AnnotatorContextException ace) {
052                ace.printStackTrace();
053                lingPipeEntityModelFile = null;
054                entityType = null;
055            }
056            System.err.println("Initializing LingPipe Entity Extractor System ...");
057            try {
058                if (entityType.equals("null")) {
059                    LingPipe_Util.initializeEntityTaggingSystem(lingPipeEntityModelFile, null);
060                } else {
061                    LingPipe_Util.initializeEntityTaggingSystem(lingPipeEntityModelFile, entityType);
062                }
063            } catch (IOException e) {
064                e.printStackTrace();
065                throw new AnnotatorInitializationException();
066            }
067            super.initialize(ac);
068        }
069    
070        public void process(JCas jcas, ResultSpecification arg1) throws AnnotatorProcessException {
071    
072            /* get document text */
073            String docText = jcas.getDocumentText();
074    
075            Iterator docInfoIter = jcas.getJFSIndexRepository().getAnnotationIndex(CCPDocumentInformation.type).iterator();
076            String docID = "-1";
077            int documentCollectionID = -1;
078            if (docInfoIter.hasNext()) {
079                CCPDocumentInformation srcDocInfo = (CCPDocumentInformation) docInfoIter.next();
080                docID = srcDocInfo.getDocumentID();
081                documentCollectionID = srcDocInfo.getDocumentCollectionID();
082            }
083    
084            if (DEBUG) {
085                System.out.println("Using LingPipe Entity Extractor on document: [" + docID + "] " + docText);
086            }
087    
088            /* run the ei system */
089            List<TextAnnotation> entityAnnotations = LingPipe_Util.extractEntities(docText, docID);
090            
091            for (TextAnnotation ta : entityAnnotations) {
092                ta.setDocumentCollectionID(documentCollectionID);
093                ta.setDocumentID(docID);
094                ta.setAnnotator(annotator);
095                Set<AnnotationSet> aSets = new HashSet<AnnotationSet>();
096                aSets.add(annotationSet);
097                ta.setAnnotationSets(aSets);
098            }
099    
100                   /* add protein annotations to the CAS */
101            UIMA_Util uimaUtil = new UIMA_Util();
102            uimaUtil.putTextAnnotationsIntoJCas(jcas, entityAnnotations);
103    
104        }
105    }
```
